# Quality of reporting randomized controlled trials (RCTs) in diabetes in Iran; a systematic review

**DOI:** 10.1186/s40200-016-0258-2

**Published:** 2016-09-07

**Authors:** Faeze Gohari, Hamid Reza Baradaran, Morteza Tabatabaee, Shabnam Anijidani, Fatemeh Mohammadpour Touserkani, Rasha Atlasi, Maryam Razmgir

**Affiliations:** 1 Knowledge Utilization Research Center, Tehran University of Medical Sciences, Tehran, Iran; 2 Endocrine Research Center, Institute of Endocrinology and Metabolism, Iran University of Medical Sciences, Tehran, 1449614535 Iran; 3 Department of Neurology, Boston Children’s Hospital, Harvard Medical School, 1 Autumn St, Boston, MA 02115 USA; 4 Endocrinology and Metabolism Research Institute, Tehran University of Medical Sciences, Tehran, Iran; 5 Iran University of Medical Sciences, Tehran, Iran

**Keywords:** Diabetes, Randomized controlled trials, Iran, Systematized review

## Abstract

**Objective:**

To determine the quality of randomized controlled clinical trial (RCT) reports in diabetes research in Iran.

**Design:**

Systematized review.

**Methods:**

We included RCTs conducted on diabetes mellitus in Iran. Animal studies, educational interventions, and non-randomized trials were excluded. We excluded duplicated publications reporting the same groups of participants and intervention. Two independent reviewers identify all eligible articles specifically designed data extraction form. We searched through international databases; Scopus, ProQuest, EBSCO, Science Direct, Web of Science, Cochrane Library, PubMed; and national databases (In Persian language) such as Magiran, Scientific Information Database (SID) and IranMedex from January 1995 to January of 2013 Two investigators assessed the quality of reporting by CONSORT 2010 (Consolidated Standards of Reporting Trials) checklist statemen.t,. Discrepancies were resolved by third reviewer consulting.

**Results:**

One hundred and eight five (185) studies were included and appraised. Half of them (55.7 %) were published in Iranian journals. Most (89.7 %) were parallel RCTs, and being performed on type2 diabetic patients (77.8 %). Less than half of the CONSORT items (43.2 %) were reported in studies, totally. The reporting of randomization and blinding were poor. A few studies 15.1 % mentioned the method of random sequence generation and strategy of allocation concealment. And only 34.8 % of trials report how blinding was applied.

**Conclusions:**

The findings of this study show that the quality of RCTs conducted in Iran in diabetes research seems suboptimal and the reporting is also incomplete however an increasing trend of improvement can be seen over time. Therefore, it is suggested Iranian researchers pay much more attention to design and methodological quality in conducting and reporting of diabetes RCTs.

**Electronic supplementary material:**

The online version of this article (doi:10.1186/s40200-016-0258-2) contains supplementary material, which is available to authorized users.

## Background

Diabetes mellitus as a chronic metabolic disorder has reached epidemic proportions globally, placing a substantial burden on healthcare services. The number of people with diabetes is growing rapidly worldwide [1, 2]. This trend even seems more in low income countries [3]. Based on the data represented by the National Survey of Risk Factors for Non-Communicable Diseases of Iran, it is estimated that 7.7 % of adults younger than 65 years had type 2 diabetes in 2008 [4].

Randomized controlled trials (RCTs) are the gold standard for evaluating new therapies or strategies in medicine [5]. However, the results of poorly designed or poorly reported RCTs can yield biased results for routine clinical practice and may impair the quality of pooled analyses such as meta-analyses [6, 7]. Therefore, the Consolidated Standards of Reporting Trials (CONSORT) statement was developed by trial methodologists and editors of biomedical journals in the mid-1990s for the explicit purpose of improving clinical trial reporting [8]. The CONSORT statement, which provides guidance to authors regarding essential items that should be included in RCT reports, was updated in 2001 (and again in 2010) to incorporate new elements [9, 10]. Although the overall quality of RCTs reporting has been improved internationally in medical sciences, to our best knowledge, there is no systematic assessment of the quality of reporting of RCTs in diabetes trials in Iran according to CONSORT.

## Methods

### Eligibility criteria

We included both parallel and cross-over RCTs conducted on diabetes mellitus in Iran. Gestational Diabetes Mellitus (GDM) trials, animal studies, educational interventions on patients with diabetes, and other studies with different designs (e.g., non-randomized controlled trials, before/after studies, quasi experimental and observational studies) were excluded. There were restrictions on English and Persian (Farsi) languages. We also excluded duplicated publications reporting the same groups of participants.

### Information sources

We identified relevant articles by a systematic search through international databases; Scopus, ProQuest, EBSCO, Science Direct, Web of Science, Cochrane Library, PubMed; and national databases (In Persian language) such as Magiran, Scientific Information Database (SID) and IranMedex from January 1995 to January of 2013 (Additional file 1).

### Quality assessment

Assessment of all included trials was carried out by two independent reviewers, and the results were entered directly into a preformatted Excel spreadsheet. Each item was assigned a yes (Y, scored as 1) or no (N, scored as 0) response depending on whether it was reported by the author and each item was weighted with equal importance. A total quality of reporting score, the CONSORT score, was calculated by simply summing the scores of the 37-item checklist, resulting in a possible range of 0–37. Thus, the maximum possible score was 37 points. Each of the study articles was then independently scored by two investigators. A final score for each item on the checklist was recorded for each article after consensus was reached through discussion between the two or in some cases, after arbitration by a third investigator. The articles were grouped by type of intervention. The main outcome was the total percentage of articles that reported each applicable section on the checklist. For clarity, the Table 2 has been depicted which 37 items would be expected to be reported. We compared the total mean number and the percentages reported of scores for each item of the CONSORT checklist between five interventions groups.

## Results

**Fig. 1:**
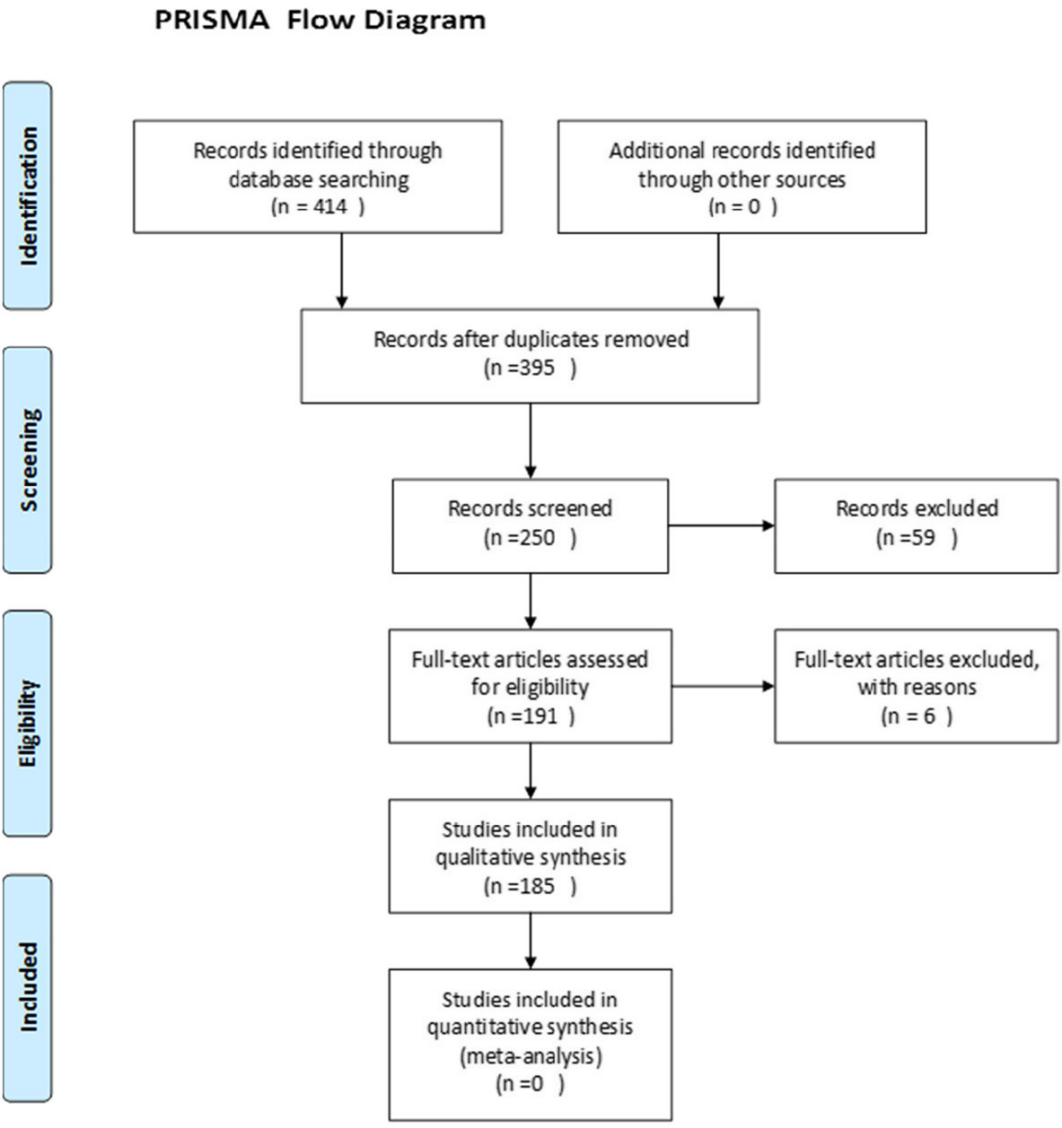
CONSORT flow diagram

Based on defined search strategy for this study, 414 articles identified. One hundred and seventy seven articles in first screening and fifty two articles in second screening were excluded. Finally, 185 articles fulfilled eligibility criteria and assessed for this systematic assessment (Fig. 1).

Table 1 shows the general characteristics of all 185 studies. The study design of 116 articles (90 %) was parallel control group trial and the rest of studies were cross over designed trial. The number of published RCTs increased from 24 articles in 2005 to 61 articles in 2012. Seventy five percent (75 %) of articles were about type 2 diabetes and the rest of studies were about either type 1 or both types of diabetes. We classified the interventions into five groups. Four major categories were pharmacological (medications), supplemental, herbal and nutritional interventions. Other interventions which performed on specific outcomes like diabetic foot or diabetic retinopathy were named as “others” group. The number of articles in each group was 54, 53, 30, 23 and 25, respectively.

**Table 1:** The characteristic of studies based on the type of interventions

		Pharmacological Number (%)	Supplemental Number (%)	Herbal Number (%)	Nutritional Number (%)	Others Number (%)	Total Number (%)
Study characteristics	Parallel	50 (92.6)	51 (96.2)	28 (93.3)	12 (52.2)	25 (100)	166 (90)
	cross-over	4 (7.4)	2 (3.8)	2 (6.7)	11 (47.8)	0	19 (10)
Year of publication	<2005	9 (16.7)	6 (11.3)	4 (13.5)	4 (17.4)	1 (4)	24 (13)
	2005–2006	7 (13)	1 (1.9)	2 (6.7)	2 (8.7)	2 (8)	14 (7)
	2007–2008	9 (16.7)	15 (28.3)	5 (16.7)	4 (17.4)	5 (20)	38 (20)
	2009–2010	13 (24.1)	14 (26.4)	7 (23.3)	3 (13)	11 (44)	48 (27)
	>2011	16 (29.)	17 (32.1)	12 (40)	10 (43.5)	6 (24)	61 (33)
Type of diabetes	Type 1	4 (7.4)	4 (7.5)	0	1 (4.3)	0	9 (5)
	Type 2	40 (74.1)	46 (86.8)	29 (96.7)	21 (91.3)	8 (32)	137 (75)
	Type 1 & 2	1 (1.9)	0	0	0	2 (8)	10 (5)
	Not mentioned	9 (16.7)	3 (5.7)	1 (3.3)	1 (4.3)	15 (60)	29 (15)
Study duration (weeks)	<1	2 (3.7)	1 (1.9)	1 (3.3)	0	0	4 (2)
	1–5	6 (11.1)	0	7 (23.3)	2 (8.7)	4 (16)	19 (10)
	5–10	10 (18.5)	22 (41.5)	14 (46.7)	12 (52.2)	3 (12)	51 (28)
	10–15	20 (37)	24 (45.3)	4 (13.3)	3 (13)	6 (24)	57 (30)
	15–20	6 (11)	2 (3.8)	3 (10)	4 (17.4)	4 (16)	19 (10)
	20–25	7 (13)	1 (1.9)	1 (3.3)	1 (4.3)	7 (28)	17 (9)
	>25	3 (5.6)	3 (5.7)	0	1 (4.3)	1 (4)	8 (4)
Estimation of sample size	<20	3 (5.6)	0	3 (10)	6 (26.1)	0	12 (6)
	20–40	12 (22.2)	10 (18.9)	2 (6.7)	4 (17.4)	4 (1)	32 (17)
	40–60	17 (31.5)	18 (34)	12 (40)	8 (34.8)	8 (32)	63 (35)
	60–80	10 (18.5)	13 (24.5)	7 (23.3)	4 (17.4)	8 (32)	42 (23)
	80–100	4 (7.4)	8 (15.1)	5 (16.7)	1 (4.3)	2 (8)	20 (11)
	>100	8 (14.8)	4 (7.5)	1 (3.3)	0	3 (12)	16 (8)
IRB approval, informed consent	Yes	53 (98.1)	53 (100)	29 (96.7)	23 (100)	24 (96)	182 (99)
	No	1 (1.9)	0	1 (3.3)	0	1 (4)	3 (1)

The sample size of these studies ranged from 8 to 282 persons with the median of 62 but most of studies (35 %) had 40 to 60 persons in their trials. The duration of studies varied depending on type of study and intervention. This duration ranged from two days to 4 years. The median RCT duration was 12 weeks and according to Table 1, the most of studies performed between 10 and 15 weeks.

Approximately all of studies (99 %) mentioned few words in their articles about patients’ informed consent or Institutional Review Board approval.

CONSORT- 2010 items according to five groups of interventions have been presented in Table 2. Title is not identified as a randomized trial in approximately in 67 % of the articles and this rate is higher in supplemental interventions (87 %). Most of the trials have provided structured summary (87.6 %), scientific background (98.4 %) and trial objectives (94.5 %) in their articles. These proportions were similar in different interventions groups. In items which were related to materials and methods section of articles (3a to 12b), description of trial design (69.7 %), eligibility criteria (97.3 %), description of interventions (93 %) and outcomes (88.6 %) and statistical methods (97.8 %) had enough description. In this part, the description of sample size calculation (21.6) and methods of additional analysis (23.7 %) were poorly reported but in nutritional interventions, additional analysis received more attention than other interventions. However reporting and describing of related items of CONSORT statement to randomization were insufficient and inadequate, only 40 % of articles had explanation about randomization. Only 33.5 % of trials have reported who was blinded after assignment to interventions. Participants and outcome assessors were blinded in 39 articles while analyst was blinded in just one trial. In addition, 36.2 % of articles mentioned description of the similarity of interventions.

**Table 2:** Quality of published articles in diabetes trials based on CONSORT items

		Pharmacological	Supplemental	Herbal	Nutritional	Other	Total
1a	yes	15 (34.9 %)	7 (13 %)	15 (50 %)	8 (48.6 %)	17 (48.6 %)	62 (33.5)
1b	yes	37 (86 %)	48 (88.9 %)	25 (83.3 %)	20 (87 %)	32 (91.4 %)	162 (87.6)
2a	yes	42 (97.7 %)	53 (98.1 %)	29 (96.7 %)	23 (100 %)	35 (100 %)	182 (98.4)
2b	yes	40 (93 %)	52 (96.3 %)	28 (93.3 %)	22 (95.7 %)	33 (94.3 %)	175 (94.5)
3a	yes	32 (74.4 %)	38 (70.4 %)	22 (73.3 %)	15 (65.2 %)	22 (62.9 %)	129 (69.7)
3b	yes	0 (0 %)	0 (0 %)	0 (0 %)	1 (4.3 %)	0 (0 %)	1 (0.5)
4a	yes	42 (97.7 %)	51 (94.4 %)	29 (96.7 %)	23 (100 %)	35 (100 %)	180 (97.3)
4b	yes	32 (74.4 %)	42 (77.8 %)	23 (76.7 %)	17 (73.9 %)	21 (60 %)	135 (73)
5	yes	40 (93 %)	46 (85.2 %)	29 (96.7 %)	22 (95.7 %)	35 (100 %)	172 (93)
6a	yes	40 (93 %)	46 (85.2 %)	26 (86.7 %)	20 (87 %)	32 (91.4 %)	164 (88.6)
6b	yes	0 (0 %)	0 (0 %)	0 (0 %)	2 (8.7 %)	0 (0 %)	2 (1.1)
7a	yes	8 (18.6 %)	9 (16.7 %)	5 (16.7 %)	9 (39.1 %)	9 (25.7 %)	40 (21.6)
7b	yes	0 (0 %)	0 (0 %)	0 (0 %)	0 (0 %)	1 (2.9 %)	1 (0.5)
8a	yes	12 (27.9 %)	5 (9.3 %)	4 (13.3 %)	2 (8.7 %)	16 (45.7 %)	39 (21.1)
8b	yes	10 (23.3 %)	10 (18.5 %)	7 (23.3 %)	2 (8.7 %)	7 (20 %)	36 (19.5)
9	yes	4 (9.3 %)	6 (11.1 %)	1 (3.3 %)	1 (4.3 %)	2 (5.7 %)	14 (7.6)
10	yes	6 (14 %)	8 (14.8 %)	2 (6.7 %)	3 (13 %)	4 (11.4 %)	23 (12.4)
11a	yes	9 (20.9 %)	18 (33.3 %)	12 (40 %)	6 (26.1 %)	17 (48.6 %)	62 (33.5)
11b	yes	10 (23.3 %)	27 (50 %)	16 (53.3 %)	5 (21.7 %)	9 (25.7 %)	67 (36.2)
12a	yes	42 (97.7 %)	54 (100 %)	29 (96.7 %)	21 (91.3 %)	35 (100 %)	181 (97.8)
12b	yes	8 (18.6 %)	12 (22.2 %)	5 (16.7 %)	10 (43.5 %)	9 (25.7 %)	44 (23.7)
13a	yes	12 (27.9 %)	6 (11.1 %)	6 (20 %)	3 (13 %)	8 (22.9 %)	35 (18.9)
13b	yes	23 (53.5 %)	26 (48.1 %)	19 (63.3 %)	14 (60.9 %)	19 (54.3 %)	101 (54.6)
14a	yes	22 (51.2 %)	21 (38.9 %)	16 (53.3 %)	6 (26.1 %)	19 (54.3 %)	84 (45.4)
14b	yes	0 (0 %)	0 (0 %)	0 (0 %)	0 (0 %)	2 (5.7 %)	2 (1.1)
15	yes	38 (88.4 %)	45 (83.3 %)	26 (86.7 %)	15 (65.2 %)	25 (71.4 %)	149 (80.5)
16	yes	14 (32.6 %)	3 (5.6 %)	8 (26.7 %)	4 (17.4 %)	13 (37.1 %)	42 (22.7)
17a	yes	15 (34.9 %)	23 (42.6 %)	9 (30 %)	6 (26.1 %)	14 (40 %)	67 (36.2)
17b	yes	0 (0 %)	0 (0 %)	1 (3.3 %)	0 (0 %)	1 (2.9 %)	2 (1.1)
18	yes	15 (34.9 %)	9 (16.7 %)	8 (26.7 %)	9 (39.1 %)	10 (28.6 %)	51 (27.6)
19	yes	22 (51.2 %)	13 (24.1 %)	13 (43.3 %)	5 (21.7 %)	26 (74.3 %)	79 (42.7)
20	yes	19 (44.2 %)	22 (40.7 %)	12 (40 %)	14 (60.9 %)	25 (71.4 %)	92 (49.7)
21	yes	5 (11.6 %)	14 (25.9 %)	10 (33.3 %)	7 (30.4 %)	13 (37.1 %)	49 (26.5)
22	yes	40 (93 %)	50 (92.6 %)	28 (93.3 %)	21 (91.3 %)	35 (100 %)	174 (94.1)
23	yes	10 (23.3 %)	12 (22.2 %)	9 (30 %)	5 (21.7 %)	5 (14.3 %)	41 (22.2)
24	yes	8 (18.6 %)	9 (16.7 %)	4 (13.3 %)	3 (13 %)	4 (11.4 %)	28 (15.1)
25	yes	22 (51.2 %)	32 (59.3 %)	19 (63.3 %)	12 (52.2 %)	7 (20 %)	92 (49.7)

Items 13a to 18 are related to results section of articles. In this section, approximately 19 % of articles used participant flow diagram. The reasons of exclusions and losses patients are explained in only 54.6 % of articles. Eighty percent of articles included a table for showing baseline demographic and characteristics for different groups. Only twenty-two percent of trials had registration numbers. Full trial protocol could be accessed only in fifteen percent and sources of funding and role of funders were mentioned only in approximately in fifty percent of trails.

## Discussion

Research in diabetes has been paid more attention in recent years. With the development of evidence-based medicine (EBM), RCTs have a high place in hierarchy level to evaluate the efficacy and safety of any trials and play an important role in decision making for clinicians. Adequate reporting of RCTs allows for easy determination of the RCT quality, which is important because RCTs of poor quality may exaggerate the effects of treatment and may potentially lead to erroneous conclusions [11–14].

To the best of our knowledge, this is the first systematize assessment of randomized clinical trials published in field of diabetes in Iran during writing this article based on CONSORT criteria. Results of the current study indicated that the quality of randomized controlled trials on diabetes needs improvement, especially in the methods section and its adherence to CONSORT guidelines seems not enough.

**Fig. 2:**
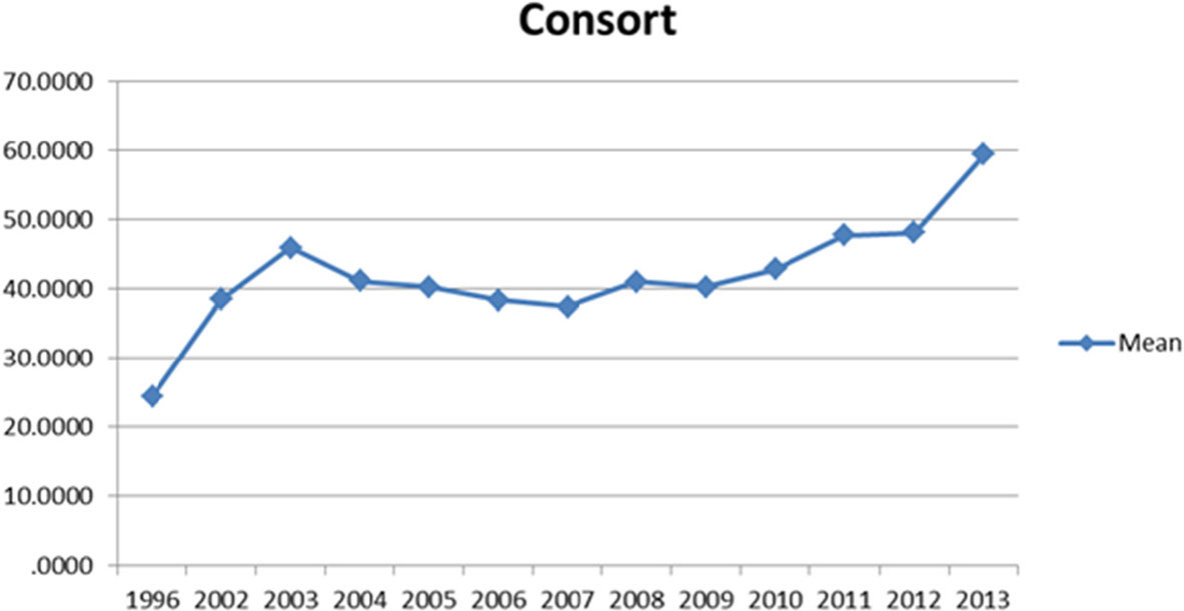
Trend of quality RCTs based on CONSORT scores between 1996–2013 years

Although there is some poor reporting of RCT in this review, it is encouraging that this study shows an increasing trend of improvement in the reporting quality of RCTs (Fig. 2). This improvement has been seen more in pharmacological and herbal medicine interventions rather than nutritional interventions.

Although we observed an overall improvement in RCT reporting over time, some methodological items such as randomization, sequence generation, sample size, allocation concealment, and blinding (29, 67, 51, 41, and 62 % of studies, respectively) remained poorly reported.

Nevertheless, adequate reporting of trial methodology is critical to avoid publication biases and to help readers decide whether the study conclusions are valid. Therefore, efforts should be made to improve the reporting of CONSORT items in future publications.

The current study has a number of limitations. Our analysis was limited to published studies, and therefore it is potentially subject to publication bias. Indeed, it is known that some RCTs, especially those with negative results, are never published. In addition, some RCTs may have been poorly designed, or manuscripts may have been so poorly written that they were rejected for publication.

Furthermore, although we report on the adequacy of reporting, as defined by the number of CONSORT-mandated items reported, we are unable to comment on the accuracy of reporting because we were unable to compare the publications to the actual trial protocols.

## Conclusion

In conclusion the findings of the current study indicated that the quality of randomized controlled trials on diabetes published in Iran needs improvement, especially in the methods section and adherence to CONSORT guidelines was not enough. Therefore, it is suggested Iranian researchers pay more attention to design and methodological quality in conducting and reporting of diabetes RCTs.

## Additional file

Additional file 1: Annex A. (DOCX 15.6 kb)

## Abbreviations

CONSORT: Consolidated standards of reporting trials
EBM: Evidence-based medicine
GDM: Gestational diabetes mellitus
RCT: Randomized controlled clinical trial
