# Supplementary material for: Quality of reporting randomized controlled trials (RCTs) in diabetes in Iran; a systematic review
Source: J Diabetes Metab Disord. 2016 Sep 7;15:36. doi: 10.1186/s40200-016-0258-2 (PMC5015251; doi:10.1186/s40200-016-0258-2)
Supplement: Additional file 1: — Annex A. (DOCX 15.6 kb) [file 40200_2016_258_MOESM1_ESM.docx]

| **Data base** | **Search Strategy** | **Results** |
| --- | --- | --- |
| **Scopus** | (TITLE-ABS-KEY(diabet* AND trial*) AND TITLE-ABS-KEY(randomized OR controlled) AND AFFILCOUNTRY(iran)) AND SUBJAREA(mult OR agri OR bioc OR immu OR neur OR phar OR mult OR medi OR nurs OR vete OR dent OR heal) AND PUBYEAR > 1994 AND PUBYEAR < 2013 AND (LIMIT-TO(DOCTYPE, "ar") OR LIMIT-TO(DOCTYPE, "cp") OR LIMIT-TO(DOCTYPE, "ip")) AND (LIMIT-TO(LANGUAGE, "English") OR LIMIT-TO(LANGUAGE, "Persian")) | 233 |
| **ProQuest** | ab(diabet* AND trial*) AND ab((randomized OR controlled)) AND iran  Date: From 1995 to 2012  Source type:Dissertations & Theses, Evidence-Based Medical Resources, Magazines, Scholarly Journals, Trade Journals  Language:English, Persian  Document type:Article, Bibliography, Conference Paper, Conference Proceeding, Dissertation/Thesis, Evidence Based Healthcare | 30 |
| **EBSCO** | Abstract: diabet* AND trial*  Abstract: randomized OR controlled  All Field: iran  1995-2012 | 4 |
| **Science Direct** | pub-date > 1994 and TITLE-ABSTR-KEY(diabet* AND trial* AND (randomized OR controlled)) and AFFILIATION(iran) | 17 |
| **Web of Science** | Title=((diabet* AND type)) AND Topic=((diabet* AND ("randomized" OR "controlled ") AND "trial*")) AND Address=("iran")  Timespan=1995-2012. | 50 |
| **Cochrane Library** | "**diabet*** in **Title, Abstract or Keywords** and **type** in **Title, Abstract or Keywords** and **(randomized OR controlled)** in **Title, Abstract or Keywords** and **trial*** in **Title, Abstract or Keywords** and **iran**, from 1995 to 2012 in **Cochrane Central Register of Controlled Trials**" | 46 |
| **PubMed** | diabet*[Title/Abstract] OR ("Diabetes Mellitus, Type 2"[Mesh] OR "Diabetes Mellitus, Type 1"[Mesh]) AND trial*[Title/Abstract] AND iran[Affiliation]  **Limits Activated:** Humans, Randomized Controlled Trial, Controlled Clinical Trial, Publication Date from 1995 to 2012 | 66 |
| **Magiran** |  | 9 |
| **SID** |  | 159 |
| **IranMedex** |  | 282 |
